# Contributions of transcriptional noise to leukaemia evolution: KAT2A as a case-study

**DOI:** 10.1098/rstb.2023.0052

**Published:** 2024-04-22

**Authors:** Cristina Pina

**Affiliations:** ^1^ College of Health, Medicine and Life Sciences, Brunel University London, Kingston Lane, Uxbridge, London, UB8 3PH, United Kingdom; ^2^ CenGEM – Centre for Genome Engineering and Maintenance, Brunel University London, Kingston Lane, Uxbridge, London, UB8 3PH, United Kingdom

**Keywords:** transcriptional noise, cell fate decisions, acute myeloid leukaemia, KAT2A, ribosomal protein genes

## Abstract

Transcriptional noise is proposed to participate in cell fate changes, but contributions to mammalian cell differentiation systems, including cancer, remain associative. Cancer evolution is driven by genetic variability, with modulatory or contributory participation of epigenetic variants. Accumulation of epigenetic variants enhances transcriptional noise, which can facilitate cancer cell fate transitions. Acute myeloid leukaemia (AML) is an aggressive cancer with strong epigenetic dependencies, characterized by blocked differentiation. It constitutes an attractive model to probe links between transcriptional noise and malignant cell fate regulation. Gcn5/KAT2A is a classical epigenetic transcriptional noise regulator. Its loss increases transcriptional noise and modifies cell fates in stem and AML cells. By reviewing the analysis of KAT2A-depleted pre-leukaemia and leukaemia models, I discuss that the net result of transcriptional noise is diversification of cell fates secondary to alternative transcriptional programmes. Cellular diversification can enable or hinder AML progression, respectively, by differentiation of cell types responsive to mutations, or by maladaptation of leukaemia stem cells. KAT2A-dependent noise-responsive genes participate in ribosome biogenesis and KAT2A loss destabilizes translational activity. I discuss putative contributions of perturbed translation to AML biology, and propose KAT2A loss as a model for mechanistic integration of transcriptional and translational control of noise and fate decisions.

This article is part of a discussion meeting issue ‘Causes and consequences of stochastic processes in development and disease’.

## 1. Transcriptional noise and cell fate decisions: initial concepts

**Figure 1.**
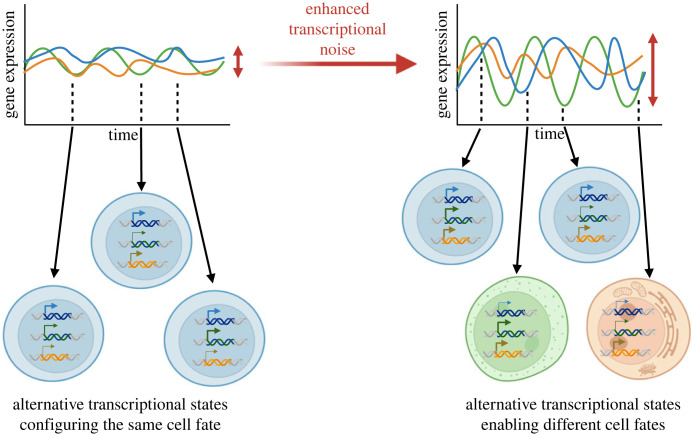
Transcriptional noise can alter cell fate decisions by allowing cells to experience alternative transcriptional states. Diagrams show time-dependent variation in the expression of three regulatory genes in an individual cell, and the impact of enhanced transcriptional noise. Expression of each gene is represented by a different colour; noise is captured by the width in gene expression levels (red double-ended arrow). Representative time-points are illustrated as snapshots of combinatorial expression of the three genes and their association with a possible cell fate, illustrated by a distinct cell colour. The colour of the loci matches the lines in time-dependent gene expression; arrow thickness indicates instant expression level. Transcriptional noise increases the number of alternative transcriptional states, increasing the likelihood of acquisition of alternative cell fates. (Figure created with BioRender.com.)

Transcriptional noise, which is defined as the time-dependent variation in the abundance of a specific transcript in an individual cell, has been proposed to facilitate cell fate changes [1–3] (figure 1). Transcriptional noise is commonly measured as coefficient of variation (CV) = standard deviation (s.d.)/mean. Alternative measures (CV^2^ = s.d.^2^/mean^2^, or Fano factor = s.d.^2^/mean) are less dependent on scaling effects of mean transcriptional levels [4], by which higher levels of noise are directly influenced by lower mean expression levels. The relative dependence between noise and mean levels results in a common perception of noise as generator of rare, low-level events, also referred to as transcriptional priming [5]. However, the correspondence between noise and low expression levels is not necessary, and it is possible for noise to be large and functionally relevant at high levels of expression.

Analysis of transcriptional noise has been performed more extensively in unicellular organisms—bacteria and yeast—as well as in viruses, where the role of noise in driving cell decisions is assumed against a background of genetic homogeneity. In these systems, modification of promoter activity resulting in transcriptional noise has been directly linked to fate changes, including proliferative behaviour [6], escape from antibiotic therapy [7,8] or entry into sporulating states [9], as a bet-hedging strategy [10–13], in face of changing environments and/or to coordinate a response to external signals [9,12,14,15], through remodelling of gene regulatory networks [13,16–18]. Baseline homogeneity is more difficult to judge in multicellular aggregates and multicellular organisms, where tissue specialization and cellular hierarchies confound the assessment of population homogeneity at the decision start point. Early work lent support to the participation of noise in mammalian cell fate decisions [19–23], associating cell fate transitions with increased molecular heterogeneity [24], albeit in a correlative manner. Nevertheless, the similarity of findings between single and multicellular organisms, including the nature of noise regulatory factors and strategies [25–27], suggests that noise participation in cell decisions is pervasive throughout the phylogenetic tree.

## 2. Transcriptional noise: tools and limitations

Transcriptional noise is by definition a dynamic property and, as such, is best analysed through repeated measurements of gene expression in the same cell, over time. Indeed, numerous studies have assessed locus-specific dynamical transcriptional activity using MS2 or PP7-based reporter systems [28,29], which can quantify and track single RNA molecules in live cells to analyse the effect of perturbations, genomic location and locus regulation [30–32] on transcriptional regimens, including noise. The systems rely on a 2-step detection of transcriptional activity, by which a fluorescent-tagged protein detects repeat loop structures engineered in the untranslated regions (UTR) of endogenous transcripts, quantifying their production. Such studies are restricted to the analysis of one or two genes and technically constrained by cellular consequences of prolonged imaging, and may not directly link transcriptional variation with functional effects, although they provide detailed analysis of transcriptional regulation.

Alternatively, fluorescent reporters of locus activity (e.g. [33–37]), which reflect engagement of the transcriptional machinery with the locus' regulatory regions and approximate the number of transcripts produced from the locus, can be used as a surrogate. Unlike MS2/PP7 systems, they do not measure endogenous transcript production. Instead, they employ the fluorescent reporter protein in lieu of the endogenous transcript to measure locus activity. They are powerful tools to detect and measure generic activity from the locus and can report the effects of specific perturbations in live cells, but they are not accurate measures of transcript-specific noise. Measurements of locus activity can be dynamic, with cell tracking, and allow correlations with cell function through either detection of morphological changes or cell separation, e.g. by flow cytometry, and downstream functional analysis. Imaging or flow cytometry measures of fluorescence can be recorded across a number of cells and the distribution of fluorescence inferred as representative of individual cell behaviour through time.

The same inference is used by single-cell measurements of RNA levels, either through imaging of fixed cells and mRNA molecule enumeration in single-molecule RNA-fluorescence *in situ* hybridization (RNA-FISH), or by RNA extraction and sequencing with quantification of aligned and normalized gene reads in single-cell RNA-sequencing (scRNA-seq). Numerous platforms and analytical algorithms for scRNA-seq have been developed over the past decade and have been extensively reviewed elsewhere [38,39]. For the purpose of this work, it is sufficient to point out that: (i) the method is destructive, therefore associations with functional states are correlative; (ii) the sensitivity of the method is dependent on the level of expression and the sequence characteristics of individual transcripts, with the potential for loss of information on certain genes or transcriptional configurations. Furthermore, scRNA-seq data are sensitive to methodology and batch effects, and it remains challenging to integrate information from different sources. Data comparisons are reliable within, but not necessarily between sequencing projects, and inferences of cell-type specific transcriptional noise levels are best performed comparatively in the same sequencing study.

## 3. Transcriptional noise: epigenetic mechanisms of regulation

**Figure 2.**
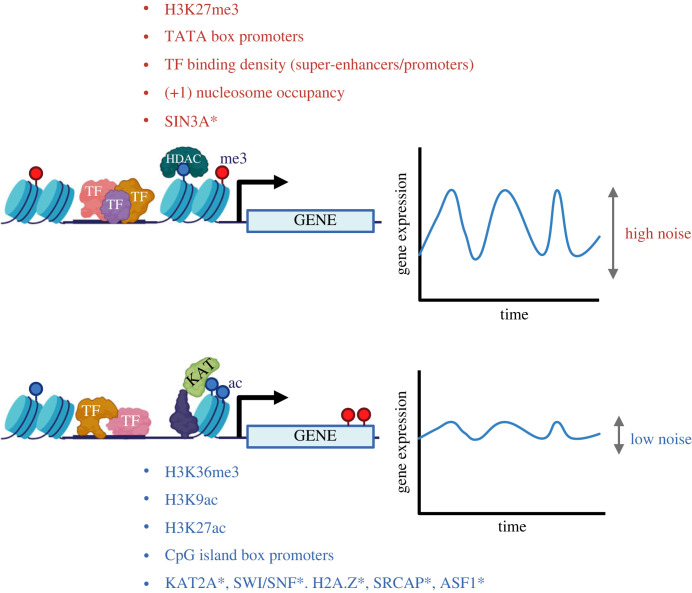
Summary of genetic and epigenetic features of locus regulation associated with transcriptional noise levels. Schematic of high and low noise in the expression of an individual gene over time, and of the main respective locus regulatory features. Noise regulatory features summarize findings in unicellular and multicellular eukaryotes as per references [25–27,41–45], which are referred to in the text. Features in red associate with high noise levels; features associated with low noise are shown in blue. *Mammalian orthologues of genes identified as noise enhancers (red) or noise buffers (blue) in yeast screens in [43]. TF, transcription factor; HDAC, histone deacetylase; KAT, lysine acetyl-transferase; me3, tri-methyl modification; ac, acetyl modification. (Figure created with BioRender.com.)

Transcriptional noise in expression of individual genes has been linked to the presence of TATA boxes [40], the density of transcription factor binding sites at promoters [41,42] and enhancers [25], and individual histone modifications [25] (figure 2). Large-scale screens in yeast [43] using fluorescent reporters of promoter activity as read-out, identified individual chromatin modifiers as modulators of transcriptional noise levels (figure 2). For the most part, these chromatin modifiers regulated histone acetylation, either directly by affecting histone lysine acetyl-transferase (KAT) activity, or indirectly by impacting non-enzymatic members of KAT complexes, as well as histone chaperones. Candidate noise enhancers such as Histone deacetylase (HDAC) complex component Rdp3s [43] have been shown to interfere with other histone marks associated with noise levels such as H3K36me3 [25,46], suggesting a structured programme of noise control. Histone acetylation is a more uniform mode of gene expression regulation in yeast when compared with mammalian systems [47,48], which have increased the variety and specificity of KAT complexes [49]. Additionally, yeast noise control is centred on promoter activity [43,44], with the emphasis shifting to enhancers in multicellular systems [25,50–53]. Nevertheless, promoter acetylation control of transcriptional noise extends to mammalian cells [27], although the specificity of individual residue acetylation may require additional investigation.

Further to increased specificity of chromatin modifier complexes and the functional significance of their respective chromatin modifications, not all chromatin modifications are pervasive throughout the evolutionary tree. For example, Polycomb repressive complex 2 (PRC2) catalyses the H3K27me3 modification associated with noise enhancement in mammalian cells [25,54], but Polycomb complexes are absent from unicellular yeast models, including colony-forming unicellular *Dictyostelium* [55]. However, the association between incipient gene repression and noise in gene expression is conserved [56], suggesting an onus on the mechanistic consequence of the chromatin activity for which the modifier may or may not be conserved.

In addition to histone modifications, histone remodellers have also been described to result in changes to transcriptional noise. Active transcription is usually associated with removal of nucleosomes at the transcriptional start site, but presence of the +1 nucleosome indicates increased noise levels [45]. Accordingly, members of the SWI/SNF complex were among the first to be described as noise modulators [44]. While the yeast subunit implicated, Snf6, does not have a mammalian orthologue [57], its submodular partners Swi3 and Snf12 correspond to mammalian Smarcc1/2 and Smarcd1–3 [58], all of which participate in stem cell specification [59,60] and tumour processes [61,62]. However, noise functions of those subunits have not been specifically addressed in yeast or mammalian cell types.

Finally, higher-order chromatin structure also impacts stability of transcription and transcriptional noise. Loss of CTCF and the associated defect in loop insulation in long-distance contacts can cause variability in transcription, both through changes in frequency of chromatin contacts and activation of transcription at the insulated locus, as well as through spurious transcriptional output from the intervening sequences [63,64]. Long-range chromatin interactions are stabilized by CTCF and cohesin in response to activation of gene expression [65], and their presence reduces variability in enhanced-promoter contacts [66] and enables state transitions [67], despite minimal effects on mean gene expression [68]. In addition to CTCF and cohesin, chromatin loops can also be established and maintained by condensin complexes [69]. Condensin's roles are predominantly in chromatin compaction during mitosis, but the presence of condensin II complexes during interphase suggests a possible role in transcriptional control [70].

## 4. Transcriptional noise: contributions to leukaemia biology

Genetic heterogeneity is central to the adaptation and evolution of cancer [71,72], with stochastic acquisition of new mutations increasing the chance of emergence of advantageous characteristics that can facilitate adaptation of subsets of cancer cells to individual environments, resulting in a dynamic sub-clonal structure. Such a pattern of stochastic evolution is akin to the putative contributions of transcriptional noise to cell state transitions [24], raising the possibility that variability in epigenetic regulation and transcriptional noise can contribute to the initiation, progression and therapeutic response of cancer cells [73–76]. Indeed, descriptive studies that investigated cell-to-cell transcriptional heterogeneity in haematological malignancies, namely chronic lymphocytic leukaemia (CLL) [77–79], associated higher levels of noise with prognosis severity [80]. Also, quantification of DNA methylation epialleles in acute myeloid leukemia (AML) upon therapy and in relapse [73,81] showed that epiallele diversification was an early event accompanying disease relapse, which preceded and was independent from acquisition of new mutations. Methylation epiallele diversification associates with transcriptional noise [81], implicating variability of gene expression levels and transcriptional programmes in cancer cell adaptation and response to therapy.

AML is the most common and deadly form of leukaemia [82]. It is dependent on epigenetic and transcriptional regulation [83,84], not only through its unique mutational spectrum targeting chromatin modifiers and transcription factors [85], but also through co-option of epigenetic regulators not specifically mutated in the disease [86]. AML has a low number of mutations in comparison with other, e.g. solid, tumours [87], reinforcing the notion that AML may rely on epigenetic and/or transcriptional events for disease evolution. Akin to genetic variegation, it is plausible that transcriptional noise produces alternative gene expression states that modify the response to existing genetic events or promote cell states capable of responding to a new set of mutations. As an illustration, the latter scenario would be compatible with accumulation of new epialleles ahead of acquisition of new mutations, as seen in therapy relapse [81]. More rarely, transcriptional noise may drive the evolution process if the acquired transcriptional state is epigenetically heritable.

Analysis of transcriptional noise levels in human leukaemia or other cancer samples is challenging as it may be confounded by heterogeneous genetic composition, which can feed into the heterogeneous single-cell transcriptomic profiles most often used to analyse transcriptional variability. For example, scRNA-seq analysis of transcriptional noise in the ageing pancreas showed a contribution of underlying mutations to the gain in noise [88]. Current methodologies for multi-omics analysis of DNA and RNA in single-cells [38] have low genomic coverage and may miss coding and non-coding mutations. Equally, not all coding mutations are detectable or even routinely investigated in single-cell RNA-seq studies of cancer samples, which limits the value of observational single-cell analysis of patient samples to address the contribution of transcriptional noise. Instead, manipulation of transcriptional noise control mechanisms provides an opportunity to address noise contributions to cancer biology in a way that is less biased by underlying genetic events. Baseline mutations will impact transcriptional variability to a similar extent in control and test cells. Moreover, deliberate introduction of oncogenic events concomitantly with the epigenetic noise perturbation makes the cancerous process less dependent on the slow acquisition of driving mutations, and minimizes the contributions of genetic variation.

## 5. Transcriptional noise: Gcn5/KAT2A as a case-study

Gcn5 is a histone acetyl-transferase responsible for acetylation of lysine 9 of histone 3 (H3K9 acetylation), which has been reproducibly associated with noise regulation in yeast [43,44]. Gene expression noise is enhanced in its absence, while elements of HDAC complexes, namely the structural component Rdp3s, have the opposite effect [43]. Moreover, loss of other elements of the SAGA complex in which Gcn5 exerts its lysine acetylation activity also enhances transcriptional noise [43], placing Gcn5 and H3K9ac as central players in noise regulation. The presence of promoter H3K9ac also correlates with reduced noise levels in mammalian cells [27], suggesting that this function may be evolutionarily conserved. Gains in transcriptional noise upon Gcn5 loss have not been specifically investigated in respect of cell decisions in yeast, but this has been addressed in mammalian cells. In mouse embryonic stem (mES) cells, we observed that KAT2A depletion results in a global gain in transcriptional noise among pluripotency and lineage differentiation genes; this associates with remodelling of gene regulatory networks, and configures a functional transition out of the pluripotent state [89].

KAT2A is one of two mammalian orthologues of yeast Gcn5, KAT2A and PCAF (also known as KAT2B), which have largely complementary expression [90]. KAT2A is the form present at early developmental stages and in mES cells, and is required for survival of mesodermal cells [91,92]. It is also the dominant orthologue in haematopoietic stem cells [93], gut epithelium [94] and in neural cells [94,95]. KAT2A is required for specific lineage fate decisions in some of these tissues [92,95–98] through acetylation of histone and non-histone proteins, including transcription factors [92,96,99], but does not perform an essential regulatory role in stem cell maintenance or differentiation. By contrast, KAT2A is specifically required for maintenance of cancer cells, namely AML [84], glioblastoma [100], gastric [101], pancreatic [102] and kidney cancer [103,104], a role that involves acetylation activity as well as deposition of other acyl-modifications [100,102].

**Figure 3.**
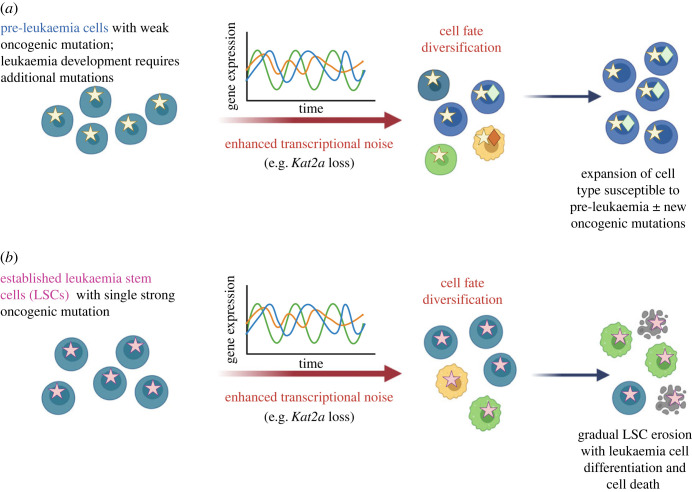
Stage-specific consequences of *Kat2a* loss and enhanced transcriptional noise for leukaemia progression. Transcriptional noise results in cellular diversification through alternative gene expression programmes leading to a higher probability of alternative cell fates (see also figure 1). (*a*) In the case of pre-leukaemia cells carrying a mutation insufficient for full leukaemia development, cellular diversification may result in the differentiation of cells susceptible to additional oncogenic mutations. Over time, these cells expand to form a propagating leukaemia clone. (*b*) In the case of well-established leukaemia cells driven by a strong oncogenic mutation, cellular diversification leads to gradual loss of propagating leukaemia stem cells, which either differentiate or die. Different mutations are represented by differently coloured star or diamond symbols. Different cell types are represented in different colours. (Figure created with BioRender.com.)

In a case-study, we used *Kat2a* loss by Mx1-Cre recombinase-dependent genetic knockout to generate *Kat2a* NULL haematopoietic stem cells and progenitors [105] in which AML was initiated through retroviral delivery of fusion oncogenes [105,106] or concomitant recombination of an oncogenic allele (e.g. *Idh1^R132H^*) [106]. As observed in mES cells, loss of *Kat2a* increased transcriptional noise [105], more markedly so in genes whose promoters lost H3K9ac, supporting a role for histone acetylation in preserving stable locus activity [107]. Downstream of the enhanced transcriptional noise, *Kat2a* NULL cells explored a wider transcriptional space, with enhanced cellular heterogeneity and increased cell differentiation outputs [105,106]. The basic observations of molecular and cellular diversification upon *Kat2a* knockout are similar to the consequences of KAT2A inhibition in mES cells [89], but the effects on leukaemia evolution are not uniform (figure 3). In the case of *KMT2A–MLLT3* AML, which constitutes a strong oncogenic event [108,109] requiring few or no additional mutations [85] to generate a rapidly progressing and aggressive leukaemia, loss of *Kat2a* has minimal consequences to disease penetrance or survival in primary leukaemia transplants, but it results in the progressive, probabilistic loss of leukaemia stem-like cells (LSCs) [105], which matches the observed exit from mES cell pluripotency [89]. LSC loss does not correspond to a scenario of full cell differentiation, but instead reflects the accumulation of incongruous cellular states [105], which result in biological dead-ends at which cells die or stop proliferating [84]. Indeed, it has been modelled that persistent transcriptional noise is incompatible with terminal cell differentiation [110], a conclusion supported by the *Kat2a* NULL data. Cellular diversification is also observed in *Kat2a* NULL pre-leukaemia states [106], but the consequences for disease progression and survival outcome are distinct. In this case, *Kat2a* NULL cells carrying the pre-leukaemic genetic event gained an advantage that allowed them to progress through the process of leukaemia transformation more efficiently than wild-type (WT) pre-leukaemia cells, resulting in higher AML penetrance and poorer survival. This may be attributed to a greater probability of generation of a transcriptional context favourable to the initiating genetic event, and/or to differential cell-state vulnerability to specific downstream mutation hits. Differentiation between the two pre-leukaemia progression scenarios requires cell barcoding experiments [111,112] to trace the trajectory and genetic composition of individual cells. Nevertheless, the scenarios are not mutually exclusive.

The two pre-leukaemia models studied by us in Gupta *et al.* [106] suggest specific dependencies or interactions between the mutational events and the gene expression programmes that respond to KAT2A loss with enhanced noise. Transcriptional noise is not sufficient to initiate the pre-leukaemia state, as *Kat2a* NULL mice do not develop myeloproliferation or indeed leukaemia [96,105]. The lack of transformation is compatible with the absence of recurrent *KAT2A* mutations in AML. Moreover, cooperation between *Kat2a* NULL-dependent transcriptional noise and an AML-associated mutation *Idh1R132H*, which does not cause AML in the absence of additional genetic hits [106,113], is also insufficient to cause leukaemia, although it confers some advantage towards leukaemia transformation *in vitro*. By contrast, *RUNX1–RUNX1T1*(exon 9a), which can drive AML with low penetrance in adult mouse models [114,115], benefits from collaboration with *Kat2a* loss to accelerate disease onset, but the benefit is lost in established *RUNX1–RUNX1T1*(exon 9a) AML cells [106]. The nature of noise-responsive transcriptional programmes may shed some light onto the dichotomy, as well as the specific cooperation between *Kat2a* loss and individual AML models.

## 6. Transcriptional noise: the nature of responsive programmes in leukaemia

Loss of KAT2A in mammalian systems [89,116], including in AML cells [93,105], affects a restricted number of genes, which contrasts with the pervasive, transcriptome-wide regulatory role of Gcn5 in yeast [47,48]. KAT2A exerts its effects in the context of two complexes—Spt-Ada-Gcn5-acetyltransferase (SAGA) and Ada two-A-containing (ATAC)—each with unique targets [93,117,118]. However, ATAC is only present in multicellular organisms, and it is not clear if and how yeast SAGA contributions to noise regulation [43] transpose to both complexes in mammalian systems. SAGA is more directly involved in regulation of tissue-specific and transcription-associated genes [93,117] that putatively make a direct contribution to cell state transitions. ATAC controls biosynthetic processes, including translation and mitochondrial metabolism [93,117]. Both complexes contribute to cancer vulnerabilities [93,119,120]: in AML, SAGA affects cell identity and differentiation status, while ATAC influences proliferation and self-renewal [93].

Unexpectedly, noise-responsive KAT2A acetylation targets are predominantly translation-associated factors and ribosomal protein (RP) genes. Although loss of KAT2A does not consistently affect mean expression levels of these genes [93,105,106], *Kat2a* NULL cells have mildly reduced protein synthesis [105,106]. Critically, reduction of protein synthesis activity in wild-type AML and pre-leukaemia cells can phenocopy some of the anti-leukaemia or pro-transformation effects, respectively, of KAT2A [105,106], tying its noise-responsiveness functions to generic biosynthetic, rather than cell-specific, genes. Furthermore, enhanced noise is observed in highly expressed genes, indicating that transcriptional noise as a biological property is not restricted to lowly or infrequently expressed genes.

RP genes have been associated with *RUNX1* mutant AML biology and they contribute to leukaemia progression [121], a phenotype compatible with pre-leukaemia acceleration. Thus, in the case of *RUNX1–RUNX1T1*, reduced translational activity may modulate responsiveness to the oncogenic stimulus. The effect does not extend to established disease [122], where *Kat2a* NULL modulation of LSC activity instead phenocopies AML dependence on high translational levels [123], both in *RUNX1–RUNX1T1* and *KMT2A–MLLT3* models.

While it is possible that other individual genes responsive to KAT2A noise modulation facilitate cellular diversification and/or modify leukaemia cell fates, consistent effects on RP gene expression and translation suggest that noise in these targets is at least partly responsible for the effects observed. Consistent with this role, aggressive CLL disease associates with noise in translation signatures, among other programmes [77], indicating a pervasive contribution to evolution of leukaemia. Other epigenetic perturbations with similar stage-specific consequences to leukaemia progression—acceleration of pre-leukaemia states [124] and differentiation of established leukaemia [125]—such as observed with *Phf19* knockout (KO), are accompanied by mild down-regulation and increased noise of RP gene signatures. In the case of KO of PRC2 component *Phf19* [124], there is also reduced noise and increased expression of self-renewal genes that lost the noise-associated H3K27me3 mark, suggesting a level of cross-talk between epigenetic and noise regulators that do not result in global modulation of variability in transcription. Independent of the H3K27me3 catalytic role, PRC2 EZH2 has been shown to positively regulate biosynthetic transcriptional programmes including RP genes [126]. And the same ribosome biosynthesis and translation-associated genes are transcriptional targets of beta-catenin and can contribute to therapy resistance in T-cell acute lymphoblastic leukaemia (ALL) [127], suggesting a pervasive role of translation in maintenance of leukaemia cell states. Beta-catenin mediates the transcriptional effects of WNT signalling, which has been suggested to preserve the existing cell state [128,129]. Wnt-mediated preservation of the cellular *status quo* is not dissimilar to the role of KAT2A [130], and may thus be exerted through buffering of transcriptional noise by interaction with the epigenetic and/or transcriptional machineries.

## 7. Transcriptional noise: modes of propagation to translational activity

Noise in transcription of RP genes can affect translational activity, either quantitatively, by varying or limiting ribosomal availability, and/or qualitatively, by modifying ribosomal composition through time. Variability in ribosomal composition and its effects on transcript-specific association with ribosomes and selective translation have gained traction as an additional way to generate heterogeneity in stem cell populations and modulate cell fate decisions. Seminal work by the Barna group [131] showed that individual ribosomal proteins (RPs), namely RPL38, participated in individual cell fate choices through specific requirement for translation of individual transcripts, in this case *Hoxa* genes. They also showed that unique ribosomal composition in ES cells differentially associates with translation of mesoderm and blood vessel development regulatory transcripts [132,133] (RPL10a-containing ribosomes) or protein synthesis in cell cycle and vitamin B12 pathways (RPS25-containing ribosomes) [132]. The relevance of all the genes and pathways affected by selective translation events to cancer biology suggests a putative contribution of ribosomal composition to tumour initiation and progression.

Several RPs are mutated in cancer and/or differentially associate with survival. Heterozygous RP mutations are causal of a subset of myelodysplastic syndromes [134,135] and ribosomal defects are central to congenital anaemias with leukaemia predisposition [136,137]. RPL22 deletion is the most commonly deleted RP in cancer, and is specifically associated with T-cell leukaemia [138]. Its paralogue RPL22L1 has two splicing isoforms (a and b) that confer distinct translational specificities, polysome abundances and disease severity in glioblastoma multiforme (GBM) [139]. However, associations of the different paralogues with survival have opposite effects in GBM and, for example, renal clear cell carcinoma, suggesting selectivity of ribosomal-dependent or -independent functions [133] or of their translational targets in tumour progression. Stage-specific effects of RPs in cancer have also been noted in breast cancer, where overexpression of a subset of RPs, including RPL15, specifically results in increased metastasis, but not primary tumour growth [140]. RPL15 enhances the translation of other RPs and of E2F-dependent signatures, putatively maintaining a positive biosynthetic loop advantageous to at least a subset of tumour cells. RPs further modify translational activity and associate with cancer survival through differential use of 5′UTR and terminal oligopyrimidine (TOP) motifs [141]. Altogether, the evidence supports stage-specific impact of perturbation of RPs and their associated translational activity in cancer. These perturbations may thus effect *Kat2a* loss through transcriptional destabilization of the translational machinery.

Translational activity is tightly controlled in haematopoietic stem cells, but the pattern is not identical in LSCs. Early work by Morrison and co-workers [123] using the *Rpl24*^min^ mouse defined a dependence of stemness on low levels of protein synthesis, with higher translational activity reducing haematopoietic stem cell activity as measured by engraftment, and promoting cell differentiation. By contrast, LSCs had higher levels of translation, and maintenance of protein synthesis activity was required for leukaemia propagation. In this and subsequent work [123,142], the authors did not specifically measure the contribution of translation to leukaemia initiation. It is possible that reduction of or instability in translational activity, either globally or of specific translational targets, including propagation through the RP repertoire [140], facilitates the acquisition of stem cell properties by differentiating cells and enables the effects of an initiating onco-mutation. As the leukaemia progresses, stable protein activity may be crucial to cell adaptation and maintenance, and its perturbation may consequently deplete LSCs. Specific epigenetic regulation of the translational machinery and the possibility of translational epimutations in AML are only starting to be addressed, and together with epigenetic and transcriptional links to proteostasis [143–146] will eventually convey an integrated DNA-to-protein function view of leukaemia regulation.

Given that KAT2A's regulation of RP gene levels impacts noise rather than mean level of expression, it is interesting to understand how the variability in transcription propagates into protein levels to change translational activity and ultimately effect cellular diversification. The prevalent understanding is that transcriptional noise is amplified at the translational level by strong translational activity [147]. In the case of KAT2A loss-mediated transcriptional noise, the prediction would be that the reduction we observed in translational activity from *Kat2a* KO pre-leukaemia [106] and AML cells [105] would buffer noise at the protein level. However, this is in contrast with the gain in cell diversity, which is a constant feature of KAT2A-depleted systems, including non-malignant stem cells [89]. Recent work has shown that the propagation of transcriptional noise into translation is nonlinear [148], with contributions from translation initiation events and 5′UTR structure [149], as well as coordination of transcriptional elongation, ribosomal loading and translational initiation for different mRNA half-lives [150], all of which can be targeted by KAT2A activity [93,105]. Alternatively, or in addition, the effects of variability can be qualitative, rather than quantitative, in terms of ribosomal assembly and transcript selectivity. Recent advances in single-cell ribosomal profiling [151] allow interrogation of cell-to-cell variability in translation, distinguishing global from transcript-specific effects and enabling the quantification of noise propagation across gene expression steps. Integration of single-cell transcriptomics, single-cell ribosomal profiling and single-cell protein measurements will bring analysis of cell heterogeneity in tumours closer to function and analysis of causality of molecular noise in cancer evolution.

## 8. Concluding remarks

The challenge remains to directly link transcriptional noise to function in malignant and developmental cell fate transitions. Analysis of KAT2A loss as a paradigm of noise control suggests that the effects are not genome-wide but specific to a subset of genes, some or all of which participate in the functional response. Perturbation of other candidate noise regulatory features, either orthologues of yeast noise regulators or individual histone modifications, ideally in similar leukaemia models, will clarify whether noise-responsive genes are in-common or specific to each regulator. Cataloguing of factor and noise-responsive genes will also (i) inform on regulatory characteristics that can be specifically engineered to modulate noise levels and (ii) identify a set of genes whose participation in regulatory networks can undergo stochastic modelling for inference and testing of effects. Application of these principles will eventually result in the development of regulation and decision-specific reporters that can directly and specifically link noise with state transitions to establish and monitor causality, and serve as biomarkers of cell fate decisions.

## Data Availability

This article has no additional data.
